# Cutaneous leishmaniasis and co-morbid major depressive disorder: A systematic review with burden estimates

**DOI:** 10.1371/journal.pntd.0007092

**Published:** 2019-02-25

**Authors:** Freddie Bailey, Karina Mondragon-Shem, Lee Rafuse Haines, Amina Olabi, Ahmed Alorfi, José Antonio Ruiz-Postigo, Jorge Alvar, Peter Hotez, Emily R. Adams, Iván D. Vélez, Waleed Al-Salem, Julian Eaton, Álvaro Acosta-Serrano, David H. Molyneux

**Affiliations:** 1 Department of Tropical Disease Biology, Liverpool School of Tropical Medicine, Pembroke Place, Liverpool, United Kingdom; 2 Milton Keynes University Hospital, Eaglestone, Milton Keynes, United Kingdom; 3 Department of Vector Biology, Liverpool School of Tropical Medicine, Pembroke Place, Liverpool, United Kingdom; 4 National Centre for Tropical Diseases, National Health Laboratory, Ministry of Health—Kingdom of Saudi Arabia, Riyadh, Kingdom of Saudi Arabia; 5 World Health Organization, Geneva, Switzerland; 6 Drugs for Neglected Disease Initiative, Geneva, Switzerland; 7 National School of Tropical Medicine, Baylor College of Medicine, Texas, United States of America; 8 Programa de Estudio y Control de Enfermedades Tropicales PECET, Universidad de Antioquia, Medellín, Colombia; 9 CBM International, Dry Drayton Road, Oakington, Cambridge, United Kingdom; 10 London School of Hygiene and Tropical Medicine, Keppel Street, London, United Kingdom; Institute of Tropical Medicine, BELGIUM

## Abstract

**Background:**

Major depressive disorder (MDD) associated with chronic neglected tropical diseases (NTDs) has been identified as a significant and overlooked contributor to overall disease burden. Cutaneous leishmaniasis (CL) is one of the most prevalent and stigmatising NTDs, with an incidence of around 1 million new cases of active CL infection annually. However, the characteristic residual scarring (inactive CL) following almost all cases of active CL has only recently been recognised as part of the CL disease spectrum due to its lasting psychosocial impact.

**Methods and findings:**

We performed a multi-language systematic review of the psychosocial impact of active and inactive CL. We estimated inactive CL (iCL) prevalence for the first time using reported WHO active CL (aCL) incidence data that were adjusted for life expectancy and underreporting. We then quantified the disability (YLD) burden of co-morbid MDD in CL using MDD disability weights at three severity levels. Overall, we identified 29 studies of CL psychological impact from 5 WHO regions, representing 11 of the 50 highest burden countries for CL. We conservatively calculated the disability burden of co-morbid MDD in CL to be 1.9 million YLDs, which equalled the overall (DALY) disease burden (assuming no excess mortality in depressed CL patients). Thus, upon inclusion of co-morbid MDD alone in both active and inactive CL, the DALY burden was seven times higher than the latest 2016 Global Burden of Disease study estimates, which notably omitted both psychological impact and inactive CL.

**Conclusions:**

Failure to include co-morbid MDD and the lasting sequelae of chronic NTDs, as exemplified by CL, leads to large underestimates of overall disease burden.

## Author summary

Cutaneous leishmaniasis is a highly prevalent vector-borne disease affecting large parts of Latin America and the Middle East, as well as parts of Northern Africa. There are several types of Cutaneous leishmaniasis, almost all of which have an active phase characterized by a disfiguring lesion (typically on exposed parts of the body), which then becomes a permanent scar (the inactive phase). We recently published an article highlighting the impact of the inactive scarring phase of CL on affected individuals, which is associated with high levels of stigma. Nevertheless, this aspect of the disease is not considered in its own right when calculating the overall disease burden by the Global Burden of Disease (GBD) Studies. In this article we estimate the prevalence of depression (major depressive disorder) in cutaneous leishmaniasis, in both the active and inactive forms. We then show the contribution of inactive CL to the overall disease burden estimates when included, which is due to the large psychological impact it has on those affected by it. We also highlight the importance of further similar efforts for other NTDs which have a chronic course, and which are also not sufficiently included in disease burden calculations at present.

## Introduction

### Cutaneous leishmaniasis

Cutaneous leishmaniasis (CL) is the most prevalent form of leishmaniasis and 1 of 22 highly prevalent neglected tropical diseases (NTD) [1]. Current disease classifications differentiate aspects of the active (nodular, ulcerative or plaque) CL lesion in terms of its transmission route (“zoonotic” vs “anthroponotic”), geographical location (“New World” vs “Old World”), and extent of its dermatological manifestations (“diffuse” vs “localised”) [2]. However, none capture the characteristic stigmatisation and psychological sequelae of life-long residual CL scarring that accompanies active infection in almost all cases. As such, we recently expanded the spectrum of CL disease by introducing new terminology—active (aCL) and inactive (iCL) scarring cutaneous leishmaniasis—to describe the dermatological changes of CL in relation to its disease activity [3]. Such a classification is also inclusive of long-term sequelae such as mucocutaneous leishmaniasis (MCL), which develops in a minority of CL cases (~4%) [4] mainly in the Americas and East African regions and which may represent a reactive form of CL [5].

The stigmatisation resulting from visible active and inactive CL lesions can be traced back centuries and was probably a major driver in establishing the ancient practice of leishmanisation [6]. Nevertheless, this defining psychosocial aspect of cutaneous leishmaniasis has been almost completely overlooked by successive disease burden studies [7–10]. Furthermore, the prevalence of inactive CL has not previously been estimated and as such is not presently incorporated into burden estimates. This unfortunately underlines a habitual lack of consideration for the chronic sequelae of NTDs. Regrettably, as CL is not a life-limiting infection, policy-makers often neglect CL as a priority disease [11–13] despite its importance to endemic communities and its links to poverty [14]. This oversight is particularly problematic given the increasing CL incidence in highly endemic conflict zones of Afghanistan, Iraq, the Syrian Arab Republic, and Yemen, creating a major public health problem [15,16].

### Major depressive disorder (“depression”)

Major Depressive Disorder (MDD) is the most prevalent form of mental disorder, affecting 4.4% of world’s population [17]. The diagnosis of MDD is symptom-based and follows the Disease Statistical Manual (DSM). MDD is one of two depressive disorders that account for the fifth largest cause of disability (years of life lived with disability; YLD) in the latest 2016 Global Burden of Disease (GBD) Study [18]. There is also a growing recognition by the global mental health community of the importance of adopting a more inclusive approach to mental health and disease, from wellness to subclinical distress to clinical “disorder”, known as the staged model of depression [19].

The psychological impact of NTDs is an area that has only recently been emphasised in the NTD community [20]. For example, mental ill health was not included in recent calculations of disability-adjusted life years (DALYs) by NTD programmes, suggesting that the psychological impact of these conditions is not a primary outcome of such programmes [21]. It is therefore unsurprising that previous global burden of depression studies appear to exclude NTDs from their prevalence and burden estimates [17,22,23] This omission is highly significant for two reasons: Many NTDs are uniquely stigmatizing [20], and collectively, WHO estimates that NTDs affect over 1 billion (or 1 in 6) people worldwide [1].

In summary, CL is often ignored at the policy level due to its lack of mortality, and is therefore a prime example of a stigmatising, prevalent NTD whose associated mental illness is disregarded. The aims of the present study are two-fold: 1) To conduct a systematic review of the psychological impact of cutaneous leishmaniasis; 2) To quantify the burden of co-morbid major depressive disorder in this highly prevalent and stigmatising condition for the first time.

## Methods

Our study reflects the current approach to disease burden estimates, which are based upon MDD as classified by the DSM [22]. We have also adopted the staged model of depression to use additional evidence from psychological and quality of life studies. These latter studies were used to calculate stages of subclinical distress associated with CL and to quantify its overall psychosocial impact.

**Fig 1:**
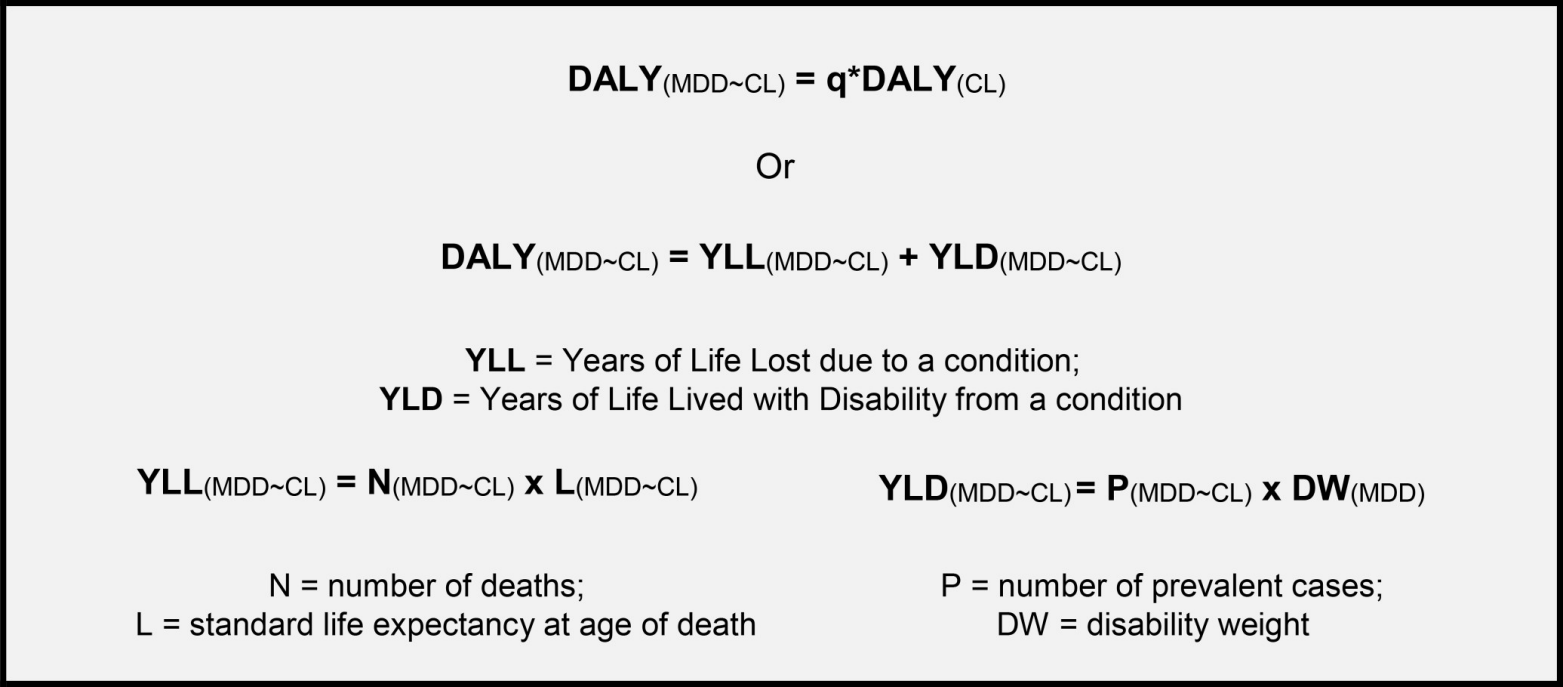
Modified disability-adjusted life years (DALY) model for calculating the burden of co-morbid conditions. Adapted from Ton *et al* (2015) [24].

There are four steps to calculating the burden of co-morbid depression (in DALYs) due to CL. Firstly, we conducted a systematic review of the psychosocial impact of all forms of CL (including MCL). To quantify the overall impact of iCL as part of the burden of CL, we also had to generate estimates of iCL prevalence for the first time. Following these first two steps, we then estimated the prevalence of MDD co-morbidity and its severity in aCL and iCL patients. We did not calculate the burden of co-morbid MCL as the associated mortality rate is not known and therefore prevalence estimates could not be reliably calculated. Finally, we multiplied the prevalence of aCL and iCL with co-morbid MDD by the disability weight (DW) for MDD at three severity levels (mild, moderate, and severe) following the methodology of Ton *et al* (2015) [24] (see Fig 1).

**Fig 2:**
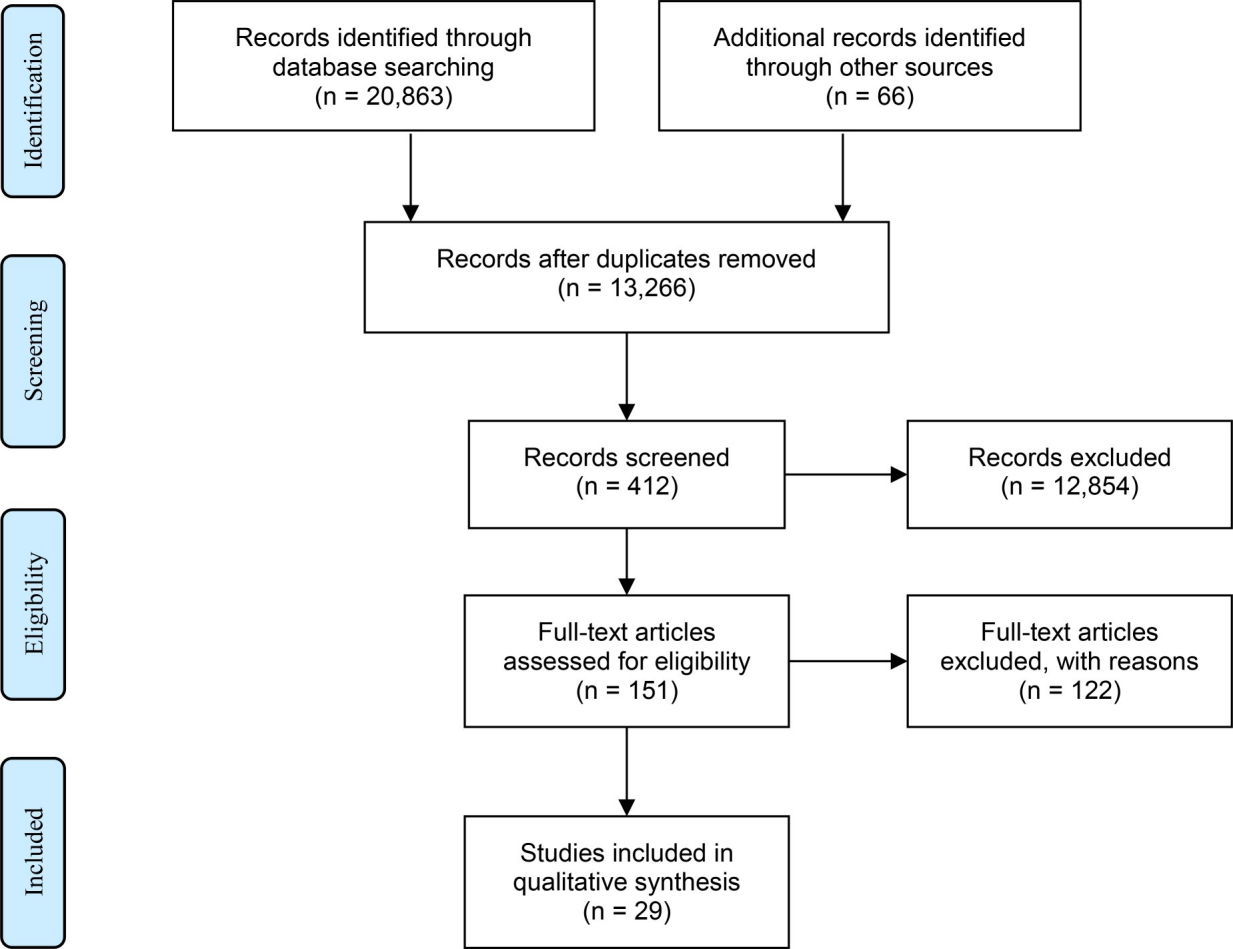
Prisma flowchart. [31].

The search strategy queried four Ovid databases–Medline [25], EMBASE [26], Global Health [27], and PSYCInfo [28]–as well as LILACS [29], using English, French, Spanish, and Portuguese search terms on 4th December 2017. Additional searches through Google Scholar [30] were performed in Arabic and English, along with back referencing of relevant articles and a grey literature search. The search strategy accounted for common terms and abbreviations for cutaneous leishmaniasis (e.g. “CL” and “cutaneous leishmaniasis”), and combined these with key words for major depressive disorder and its symptoms, as well as general psychological impact (e.g. “psych*”, “major depressive disorder”, “distress”). We included all relevant psychological studies in CL patients and those with reliable knowledge of their experiences (i.e. their caregivers and their care providers) (Fig 2). As such, community studies were excluded from our final analysis except to further contextualise our findings. Please see S1 Appendix for further details of the search strategy and individual terms queried. Please see S2 Appendix for our inclusion and exclusion criteria, and S3 Appendix for the reasons for excluding studies from final analysis.

## Results

### Estimating major depressive disorder co-morbidity in cutaneous leishmaniasis

Twenty-nine studies were included in the final analysis of the psychosocial impact of CL (see S4 Appendix). The large majority (25/29) of studies were based in middle-income countries (18/29 UMIC, 7/29 LMIC) [32]. Similarly, most studies took place in the highest burden world regions (12/29 in the Eastern Mediterranean Region (EMR) and 11/29 in the Americas Region (AMR)), and included 11 of the 50 highest burden countries for CL in the world [9].

Studies that quantified an MDD diagnosis or symptoms using both validated (e.g. SCID-1; BDI) and unvalidated tools (e.g. self-reported depression symptoms) were used to determine rates of co-morbid MDD in both aCL and iCL (See Table 1). Additional quality of life, stigma, socioeconomic, and qualitative studies were used to generate an estimate of subclinical “distress” as per the staged model of depression (see Tables 2 and 3).

**Table 1:** Quantitative studies: MDD diagnosis and symptoms (validated and non-validated measures).

Author (Year)	Country (WHO Region)	Economic Development	Sample size	Sex	Age	Lesion location	Disease activity	MDD measure	Results	Interpretation
**MDD diagnosis: Validated measure**										
**Simsek *et al*** **(2008) [33]**	Turkey (EUR)	UMIC	64 (8 CL; 56 non-CL primary care)	100% F (Overall)	30 (Overall)	-	aCL	SCID-I (mental disorder)	CL: 53.3% Non-CL: 24.1%	aCL: independent risk factor for mental disorder; MDD most prevalent mental disorder; Multiple mental disorders common
**Torkashvand *et al* (2016) [34]**	Iran (EMR)	UMIC	160 (80 iCL; 80 former CL, no iCL)	43.1% F (Overall)	31.01 mean (Overall)	50% face; 50% rest of body	iCL	BDI	BDI (mean): Face: 11.66 ± 15.38 Body: 11.64 ± 14.11 No scar: 3.82 ± 8.09	0–13: Minimal 14–19: Mild 20–28: Moderate 29–63: Severe [35] BDI severity: Face: mild = 12.2%; mod = 4.9%; sev = 17.1%) Body: mild = 7.7%; mod = 15.4%; sev = 12.8% No scar = 3.8%; mod = 6.3%; sev = 2.5%
**Turan *et al*** **(2015) [36]**	Turkey (EUR)	UMIC	94 (54 CL; 40 healthy controls)	54% F (CL); 50% F (Control)	7–12; 13–18	65% face	aCL	CDI (Also QoL; see below)	7–12 (mean): aCL: 9.72 ± 6.11 Control: 4.5 ± 3.83 8–13 (mean): aCL: 14.25 ± 4.76 Control: 4.50 ± 2.46	Clinical cut-off: 13 Community cut-off: 19 [37] 8–13: Over 50% met clinical cut-off; those within 1 SD of mean met community cut-off. Patients aged 7–12: Those within 1 SD of mean met clinical cut-off; those within 2 SD met community cut-off Significantly higher than control at both age groups
**Yanik *et al*** **(2004) [38]**	Turkey (EUR)	UMIC	99 (33 iCL; 33 aCL; 33 healthy controls)	50.5% F (Overall)	18 (Overall)	70% face 30% UL (Overall)	aCL/ iCL	HADS-D (Also QoL; see below)	Mean aCL: 7.24 ± 3.91 Mean iCL: 8.67 ± 3.83 Mean control: 5.76 ± 4.01	Mild: 8–10 Moderate: 11–14; Severe: 15–21 [39] aCL: Mean score on cusp of mild MDD; those within 1 SD of mean met cut-off for moderate MDD iCL: Over 50% met cut-off for mild MDD; those within 1 SD of mean met cut-off for moderate MDD Depression rates significantly higher CL vs control
**MDD symptoms: Validated measure**										
**Honório *et al* (2016) [40]**	Brazil (AMR)	UMIC	44	54.5% F	51.8	-	aCL	WHO-QoL Bref	Q26 Negative feelings (blue mood, anxiety, despair, depression) 90.9%	Frequency: 18.18% always; 43.18% very often; 20.45% quite often; 9.09% seldom; 9.1% never
**Hu *et al*** **(2015) [41]**	Suriname (AMR)	UMIC	163	8.3% F 7.6% F	7 day: 33 (median) 3 day: 30 (median)	7 day: 10.7% face 13 day: 7.7% face	aCL	EQ-5D (Also QoL; see below)	Depression/Anxiety: Pre-treatment: 50.6% (7 day and 3 day) Post-treatment (6 wks): 7 day: 2.9; 3 day: 9.4%	Significantly reduced symptoms of anxiety and depression with treatment (both 3- and 7-day regimes)
**Torkashvand *et al* (2016) [34]**	Iran (EMR)	UMIC	160 (80 iCL; 80 former CL, no iCL)	43.1% F (Overall)	31.01 mean (Overall)	50% face; 50% rest of body	iCL	BSQ	BSQ (mean): Face: 4.73 ± 9.59 Body: 5.89 ± 8.91 No scar: 1.22 ± 3.47	Suicide risk level: Face: never = 78.0%; low = 4.9%; high = 17.1% Body: never = 61.5%; low = 12.8%; high = 25.6% No scar: never = 87.5%; low = 10.0%; high = 2.5%
**MDD diagnosis/symptoms: Non-validated measure**										
**Al-Kamel (2017) [42]**	Yemen (EMR)	LMIC	11	90.91% F	29.4 (mean)	38.5% UL 61.5% face	aCL 72.7% iCL 18.2% MCL 9.1%	Depression	27.3%	Self-reported depression rates not affected by presence of MCL
**Bastidas *et al* (2008) [43]**	Venezuela (AMR)	UMIC	17	58.8% F	25–34 mode	-	aCL/ iCL	Low mood	Total: 58.8% F: 100% M: 0%	Marked sex difference
**Pacheco *et al* (2017) [44]**	Brazil (AMR)	UMIC	24	62.5% F	38.8	100% exposed	aCL	Sadness, depression, low mood	Total: 12.5% (F: 33.3%; M: 0%)	Marked sex difference
**Semeneh (2012) [45]**	Ethiopia (AFR)	LIC	10	50% F	29.3	100% face	aCL 30% iCL 70%	High depression	30%	

BDI = Beck Depression Inventory; BSS = Beck Scale for Suicidal Ideation; CDI = Children’s Depression Inventory; EQ-5D = Euro-Qol-5 Dimensions; HADS-D = Hospital Anxiety and Depression Scale–Depression; LIC = Low Income Country; LMIC = Lower-Middle Income Country; SCID-I = Systematic Clinical Interview for Depression– 1^st^ version; UMIC = Upper-Middle Income Country; WHO-QoL Bref = World Health Organization Quality of Life Short; UL = upper limbs

**Table 2:** Quantitative studies: Quality of life, psychological distress, stigma, socioeconomic impact studies.

Author (Year)	Country (WHO Region)	Economic Development	Sample size	Sex	Age	Lesion location	Disease activity	Measure	Results	Interpretation
**Quality of life**										
**Chahed *et al* (2016) [46]**	Tunisia (EMR)	LMIC	41	100% F	85% <30	93% face 54% rest of body	iCL	WHO-QoL Bref	Social relationships: 63.0 Psychological: 52.6 Social: 61.8 Environmental: 47.8 Total: 56.3	Psychological QoL on the verge of significant correlation with PLSI (p <0.087)
**Elsaie *et al* (2017) [47]**	Egypt (EMR)	LMIC	12	16.7% F	32	- (almost all exposed)	aCL	DLQI	Pre-treatment: 12.67 Post-treatment: 4.25	Pre-treatment: very large impact [48] Post-treatment: small impact [48] Significant reduction in all domains of QoL
**Handjani *et al* (2013) [49]**	Iran (EMR)	UMIC	50 (5 CL; 10 Psoriasis; 15 Vitiligo; 20 Pemphigus)	54% F (Overall)	42 (Overall)	**-**	aCL	FDLQI	Mean CL: 12.00 ± 4.80 Mean Psoriasis: 14.70 ± 5.01 Mean Vitiligo: 14.40 ± 5.08 Mean Pemphigus: 15.45 ± 4.70	Main concern (CL) is time spent looking after partner/relative (40%)
**Honório *et al* (2016) [40]**	Brazil (AMR)	UMIC	44	54.5% F	51.8	-	aCL	WHO-QoL Bref	Social relationships: 74.62 Psychological: 70.55 Physical: 61.85 Environment: 59.80 Total: 66.70	Q1-2: 81.82% rated QoL good or very good
**Hu *et al*** **(2015) [41]**	Suriname (AMR)	UMIC	163	8.3% F 7.6% F	7 day: 33 (median) 3 day: 30 (median)	7 day: 10.7% face 13 day: 7.7% face	aCL	Skindex-29	Pre-treatment 7 day: 28.4 3 day: 31.0 Post-treatment 7 day and 3 day: 1.7	Significant improvement post-treatment in both 3- and 7-day trials Mean pre-treatment score for both cohorts equates to moderate QoL impact (25–49.9) [50]
**Nilforoushzadeh *et al* (2010) [51]**	Iran (EMR)	UMIC	80	100% F	- (>10)	-	aCL	DLQI	Drug + Psychotherapy Before: 10.6 ± 5.7 After: 7.7 ± 4.6 Drug alone Before: 10.0 ± 5.1 After: 11.0 ± 5.1	Before treatment QoL: No impact: 0% Small impact: 26.125% Moderate impact: 46.125% Very large impact: 25.0% Extremely large impact: 2.5%
**Ranawaka *et al* (2014) [52]**	Sri Lanka (SEAR)	LMIC	146	28% F	31 (median)	45% UL 25% face 20% LL 10% trunk	aCL	DLQI	Mean: 5.58	Severity: No impact: 21.0% Small impact: 34.2% Moderate impact: 30.8% Very large impact: 13.3% Extremely large impact: 0.7%
**Toledo *et al* (2013) [53]**	Brazil (AMR)	UMIC	20	15% F	45.6	40% exposed areas	aCL	DLQI	Mean: 9.75	Severity: No impact: 0% Small impact: 30% Moderate impact: 30% Very large impact: 40% Extremely large impact: 0%
**Turan *et al*** **(2015) [36]**	Turkey (EUR)	UMIC	94 (54 CL; 40 healthy controls)	54% F (CL); 50% F (Control)	7–12; 13–18	65% face	aCL	PedQoL	7–12 aCL: 81.31 ± 11.39 Control: 91.83 ± 4.76 13–18 aCL: 74.99 ± 13.95 Control: 80.34 ± 4.74	Young aCL patients significantly worse QoL than controls Parents’ QoL scores also significantly lower than controls
**Vares *et al* (2013) [54]**	Iran (EMR)	UMIC	124	62.9% F	36.9 (mean)	70% UL 15% face 10% LL	aCL (94%) iCL (6%)	DLQI	5.87 ± 5.96	Severity: No impact: 26.8% Small impact: 30.5% Moderate impact: 24.2% Very large impact: 15.3% Extremely large impact: 3.2%
**Yanik *et al*** **(2004) [38]**	Turkey (EUR)	UMIC	99 (33 iCL; 33 aCL; 33 healthy controls)	50.5% F (Overall)	18 (Overall)	70% face 30% UL (Overall)	aCL/ iCL	DQLI	aCL: 34.77 ± 8.47 iCL: 24.11 ± 8.56	QoL better in aCL vs iCL Moderate correlation with HADS-D and DQLI (r_s_ = 0.291)
**Psychological distress**										
**Bennis *et al* (2017) [55]**	Morocco (EMR)	LMIC	86	42.2% F	17.7	-	aCL/ iCL	Psychosocial impact	“Yes” = 48.8% “Maybe” = 40.7% “No” = 10.5%	Somewhat town-dependent: 13% “no psychosocial impact” in one town; 7% “no psychosocial impact” in another town
**Chahed *et al* (2016) [46]**	Tunisia (EMR)	LMIC	41	100% F	85% <30	93% face 54% rest of body	iCL	PLSI	PLSI: 9.5 ± 6.7	A score of 10+ denotes a high degree of stress in psoriatic patients [56]
**Stigma, disfigurement, and socio-economic impact**										
**Al-Kamel (2017) [42]**	Yemen (EMR)	LMIC	11	90.91% F	29.4	38.5% UL 61.5% face	aCL 72.7% iCL 18.2% MCL 9.1%	Stigma	Social: 63.64% Aesthetic: 63.64% Psychological: 72.73%	1+ forms of stigma: 90.91% (1 form: 27.27% 2 forms: 18.18% 3 forms: 45.45%)
**Abazid *et al* (2012) [57]**	Syria (EMR)	LMIC	70	59% F	32.3	-	aCL/ iCL	Disfigurement	32.9%	Worst effects of CL: Appearance of aCL (68.6%) and permanence of iCL (32.9%)
**Chahed *et al* (2016) [46]**	Tunisia (EMR)	LMIC	41	100%F	85% <30	93% face 54% rest of body	iCL	Exclusion Body image Worse marital prospects	73% 58% 75% (for M); 59% (for F)	
**Fernando *et al* (2010) [58]**	Sri Lanka (SEAR)	LMIC	120	27% F	31.6	F: 56% face M: 41% UL	aCL	Isolation and social stigma Absent/unable to work	18% M 25% F 55% M; 40% F	Worse with facial lesions
**Pacheco *et al* (2017) [44]**	Brazil (AMR)	UMIC	24	62.5% F	38.8	100% exposed	aCL	Social discrimination Family discrimination	Total: 37.5% (F 66.6%; M 20%) Total: 20.8% (F: 55.5%; M 0%)	Marked gender differences
**Ramdas *et al* (2016) [59]**	Suriname (AMR)	UMIC	205	10.7% F	30–39 mode	- (face rare)	aCL	Shame, disgust Enacted stigma	18.5% 16%	Author reports low stigma due to rarity of facial lesions
**Reithinger *et al* (2005) [60]**	Afghanistan (EMR)	LIC	83 (parents of affected)	100% F	-	-	aCL/ iCL	Disfigurement	54% felt disfigured children by lesions/scars, treatment, exclusion	
**Ruoti *et al* (2013) [61]**	Paraguay (AMR)	UMIC	25	28% F	49	-	CL/ MCL	Shame	12.5%	
**Semeneh (2012) [45]**	Ethiopia (AFR)	LIC	10	50% F	29.3	100% face	aCL 30% iCL 70%	Disgrace/ despair Shame Low self-esteem, guilt	80% 40% 70%	
**Weigel *et al* (1994) [62]**	Ecuador (AMR)	UMIC	208	46.6% F	35.8	-	aCL/ iCL	Impact on ability to work Low self-esteem	iCL: 68.9% aCL: 61.3% Total: 67.1% iCL: 82.7% aCL: 76.9% Total: 81.3%	Men significantly more than women Woman significantly more than men
**Yanik *et al*** **(2004) [38]**	Turkey (EUR)	UMIC	99 (33 iCL; 33 aCL; 33 healthy controls)	50.5% F (Overall)	18 (Overall)	70% face 30% UL (Overall)	aCL/ iCL	BIS	aCL: 17.15 ± 11.07 iCL: 21.0 ± 8.16 Control: 38.69 ± 6.37	Body image significantly reduced; moderately correlated with HADS-D (r_s_ = 0.256)

DLQI = Dermatology Life Quality Index; DQLI = Dermatology Quality of Life Index; FDLQI = Family Dermatology Life Quality Index; PedQoL = Pediatric Quality of Life; PLSI = Psoriasis Life Severity Index; UL = upper limbs; LL = lower limbs

**Table 3:** Qualitative studies.

Author (Year)	Country (WHO Region)	Economic Development	Sample size	Sex	Age	Lesion location	Disease activity	Results
**Al-Kamel (2017) [38]**	Yemen (EMR)	LMIC	11	90.91% F	29.4	38.5% UL 61.5% face	aCL 72.7% iCL 18.2% MCL 9.1%	Fear and social isolation common; Oldest patient (60yo) suggests stigma is age-related; Concerns about facial lesions and marital prospects
**Alorfi (2016) [63]**	Saudi Arabia (EMR)	HIC	21 (Health Workers; HW)	42.86% F	-	-	aCL/ iCL	Stigma from iCL noted by 8/21 HWs; 1/21 says no stigma Parental guilt at children being affected; All HWs agree CL has psych impact; main concern is iCL; 1/21 HW recounts suicidal ideation in patient; Low self-esteem and depression common; Fears regarding lack of effective treatment
**Bennis *et al* (2017) [55]**	Morocco (EMR)	LMIC	86	42.2% F	17.7	-	aCL/ iCL	Low self-esteem and diminished social value common; Marital prospects decreased; psychological impact can increase after treatment as scar remains; fear and worry concerning lack of treatment services
**da Silva *et al* (2004) [64]**	Brazil (AMR)	UMIC	8	100% F	**-**	**-**	aCL	Fearful for health; uncertain of treatment; feel trapped; worried about appearance
**Guevara *et al* (2007) [65]**	Venezuela (AMR)	UMIC	30 (Dermatologists, health inspectors/promoters, nurses, social workers)	-	-	-	aCL	All participants: patients express cultural significance of aCL and its psychological impact, but this is not registered by healthcare professionals due to strictly disease-focused, biomedical approach to CL Perception is location dependent. CL is seen either as a “sore”, “leprosy”, or a “bite”. Differential impact depending on how it is perceived.
**Martins (2014) [66]**	Brazil (AMR)	UMIC	7	20% F	45	**-**	aCL	Strong social impact of aCL and iCL on work, church and school Fear, low self-esteem, depression, and isolation frequently seen
**Ramdas *et al* (2016) [59]**	Suriname (AMR)	UMIC	205	10.7% F	30–39 mode	- (face rare)	aCL	Social restrictions infrequent due to cohesiveness of local community and recognition of CL as a non-contagious disease
**Reyburn *et al* (2000) [67]**	Afghanistan (EMR)	LIC	84	54.8%	28	- (usually face/hands)	aCL	Males more affected in work and public life (religion, work), females more affected at home (cooking, hospitality); overall equal impact Most report stigmatisation; in some, strong feelings of shame Need to isolate CL sufferers developed into personal rejection; lack of personal contact particularly problematic for children Very rare for CL to stimulate more caring attitudes towards sufferers
**Semeneh (2012) [45]**	Ethiopia (AFR)	LIC	10	50% F	29.3	100% face	aCL 30% iCL 70%	MDD symptoms (low self-esteem, hopelessness, sadness) very common; Poor QoL due to CL impact on SES, and lack of treatment services; Main concern is aCL, yet all left disfigured by iCL scar; Commonly insulted with local terms for both iCL and aCL; Vast majority experienced stigma, especially in aCL phase; Unaffected people favoured for work, especially if facial lesions; Marital rejection common, though some believe not a problem

AFR = African Region; AMR = Region of the Americas; EMR = Eastern Mediterranean Region; EUR = European Region; HIC = High Income Country; SEAR = South-East Asian Region

A diagnosis of MDD was consistently reached within the mean or one standard deviation of the mean in CL patients [33,34,36,38], equating to MDD rates of 30–50%. Meanwhile, quantification of symptoms of MDD mostly relied upon self-reporting. As such, symptoms of low mood and depression in CL patients ranged from 12.5–90.9% [34,40–45] aCL patients had significantly higher rates of MDD compared to controls in both children and adults [36] aCL was also found on multivariate analysis to be an independent risk factor for mental disorder in the primary care setting [33]. It is therefore unlikely that these results are a product of significant selection bias.

Equally, whilst rates of MDD were not measured for children with iCL, significantly higher rates of MDD were found in adults compared to controls. iCL patients were also at significantly higher suicide risk than controls [34]. In the only study to measure co-morbid MDD in both aCL and iCL, CL scarring was associated with non-statistically significantly higher MDD scores [38]. These findings are important, as considerably more patients are in the inactive (scarring) phase of CL than in the active phase. Although the data suggest that rates of MDD in iCL are at least equal to those found in aCL patients, the majority of studies (16/29) focused exclusively on aCL.

More broadly, quality of life was found to be significantly decreased in CL patients compared with controls. Stigma was a characteristic feature of CL in most quantitative and qualitative studies, whilst psychological distress was found to be between 50–90% [46,55]. Similarly, issues of disfigurement and reduced capacity to work affected the majority of sufferers (see Table 2). Interestingly, the psychological burden extended to CL caregivers, who were also found to have significantly elevated depression rates [36] and diminished quality of life [36,49] compared to controls.

Overall, CL is associated with a high degree of psychological morbidity irrespective of country, age, and disease activity. We present two other important patient- and disease-specific variables considered during our analysis: patient sex and lesion location. These were chosen due to multiple reports linking them with increased psychosocial impact. Indeed, despite findings of qualitative studies that facial lesions are the most psychologically damaging [42,45,63,67], none of the four quantitative studies [34,46,52,54] providing subgroup analysis demonstrated a statistically significant association with facial lesions and worsening psychological outcomes. Moreover, facial iCL scars were actually associated with lower rates of depression and suicidality than those located on other parts of the body [34]. Instead, it may be more appropriate to differentiate the visibility of lesions in future studies.

A significant number of studies focused solely on women (5/29) on the basis that women are generally at greater risk of depression [17]. It is therefore important to consider possible sex differences in MDD rates given that men have more reported cases of CL than women in most endemic countries [4] Interestingly, women-only studies were found to have comparable MDD rates to mixed sex studies, although differences in self-reported symptoms of MDD were noted in some countries [43,44]. The reasons for these findings could perhaps be explained by community [68], socio-economic [62], and qualitative studies [67]. For example, whilst women are commonly more concerned by bodily appearance and marital prospects, a roughly equal impact is placed upon men through incapacity to work and perform leadership responsibilities [52] due to the disease.

Based on the available evidence, we conservatively estimate that 70% of individuals with both active and inactive CL will experience some degree of psychological morbidity. This ranges from subclinical “distress” (50%) to clinical “disorder” (20%), in accordance with the staged model of depression [19] As such, 30% of CL patients fall into the “wellness” category of the model, in view of regional differences in psychosocial impact [55,65] and the small number of countries and endemic communities in which CL is less stigmatizing [59] and perceived as less severe [69] (see Table 4).

**Table 4:** Estimating the psychological impact of CL using the staged model of depression adapted from Patel (2017) [19].

Stage	Definition	CL estimate	References
**Wellness**	Absence of any sustained, distressing, emotional experiences	**30%**	[57–59, 61]
**Distress**	Mild to moderate distressing emotional experiences of relatively short duration	**50%**	[46, 47, 49, 51–55, 60, 62]
**Major Depressive Disorder**	Severely distressing experiences, lasting at least two to four weeks, with impairment of social functioning	**20%**	[33, 34, 36, 38, 40–45]
**Recurrent Major Depressive Disorder**	Unresponsive or relapsing depressive episodes		

### Calculating the prevalence of inactive CL

The 2016 GBD Study provides CL prevalence estimates that account solely for aCL and that also include MCL within them unseparated. As such, the prevalence of inactive (scarring) CL has not been previously estimated, and is not incorporated formally into the GBD burden estimates for CL. The methodology for calculating the prevalence of inactive CL has been previously described [3]. In short, our calculations are derived from the latest reported aCL incidence data from WHO spanning 2006–2015 [70] that have been adjusted for underreporting [10,71] and the presence of MCL within them [72–74] (see Table 5). We assume zero CL-associated mortality and a life expectancy of 30 years with scarring; this is a conservative longevity estimate considering the life expectancy of at-risk populations in high burden countries [74]. For further information on this methodology, please see S5 Appendix.

**Table 5:** Estimating the prevalence of inactive CL.

	Active CL *(GBD 2016)* [18]	Inactive CL	Total
**Ratio**	~10	~90	100
**Prevalence**	4,320,000	33,883,900	38,203,900

### Estimating the severity of major depressive disorder co-morbidity in cutaneous leishmaniasis

GBD Studies differentiate the severity of episodes of MDD at three levels—mild, moderate, and severe–each with its own disability weight [22]. Therefore, it is necessary to calculate the severity of co-morbid MDD in CL patients to calculate the disability burden (YLD) component of the DALY.

In the studies we identified, the mean depression scores of CL patients equated to mild MDD, with moderate MDD scores being reached within one standard deviation in most studies. Furthermore, in a study of depression in inactive CL using Beck’s Depression Inventory, ~70% of cases with depression scored in “mild” severity [34]. Due to the relatively small sample sizes and difficulties in comparing MDD severity from different measurement tools, we used data from the 2010 GBD study on depressive disorders to help inform our estimates (see Table 6). In that study, the patient MDD cohort was classified accordingly: 72.7% with *Mild* severity; 16.5% with *Moderate* severity; and 10.8% with *Severe* MDD [22].

**Table 6:** Estimating the severity of co-morbid MDD in cutaneous leishmaniasis.

Severity of MDD	Disability Weight^75^	Severity of MDD In 2010 GBD Study [22]
Mild	0.145	72.7%
Moderate	0.396	16.5%
Severe	0.658	10.8%

### Quantifying DALYs for major depressive disorder in cutaneous leishmaniasis

Applying the estimate for MDD severity to our prevalence estimates for cutaneous leishmaniasis, the following YLDs were calculated: 200,000 for active CL, and 1.7 million for inactive CL (combined total 1.9 million YLDs for CL) (see Table 7 and Table 8). We assumed no mortality burden associated with MDD co-morbid to cutaneous leishmaniasis, and as such our YLD figures equalled the overall DALY figures (see S6 Appendix for in-depth calculations). These figures only represent the impact of co-morbid MDD in this condition and do not account for the impact of other mental disorders such as anxiety disorders or the subclinical state of distress as per the staged model of depression [19].

**Table 7:** Estimating the burden of Major Depressive Disorder in cutaneous leishmaniasis.

	Active CL*	Inactive CL	Total CL
**Prevalence**	4,320,000 [18]	33,883,900	38,203,900
**Prevalence with MDD** *(%)*	20%	20%	20%
**Disability Weights** *(GBD 2016)* [75]	0·145 (Mild MDD) 0.396 (Moderate MDD) 0.658 (Severe MDD)	0.145 (Mild MDD) 0.396 (Moderate MDD) 0.658 (Severe MDD)	0.145 (Mild MDD) 0·396 (Moderate MDD) 0.658 (Severe MDD)
**YLDs** *(Co-morbid MDD alone)*	208,932	1,687,065	1,895,997
**YLLs** *(Co-morbid MDD alone)*	0	0	0
**DALYs** *(Co-morbid MDD alone)*	208,932	1,687,065	1,895,997

**Table 8:** Overall DALY estimates for cutaneous leishmaniasis (aCL and iCL).

	Active CL	Inactive CL	Total
**Physical health DALYs** *(GBD 2016)*^*76*^	273,000*	-	273, 000
**Co-morbid MDD DALYs**	208,932*	1,687,065	1,895,997
**Physical health +** **Co-morbid MDD DALYs**	481,932*	1,687,065	2,168,997

*GBD estimate includes MCL

## Discussion

The results presented here challenge the most recent GBD estimates for the overall burden of CL given the prevalence of mental illness reported in the literature for the condition. We highlight the lack of reliable prevalence estimates on which GBD figures are based. We further emphasise that, despite the increased recognition of NTDs through their inclusion within the UN Sustainable Development Goal (SDG) health targets, the burden of mental health associated with stigmatising and chronically disabling NTDs is not appropriately factored into the calculations of overall global mental health estimates. We stress the importance of residual disease on the continuing suffering of those with NTDs using the example of inactive CL.

Indeed, inclusion of iCL increases the CL prevalence estimate 10-fold, which substantially increases the CL disease burden in itself. However, factoring in the burden of co-morbid MDD for both aCL and iCL further increases its overall burden to 2.2 million DALYs. This is approximately eight times greater than the previous DALY estimate reported in the 2016 GBD study that accounted for aCL alone [76]; this is despite our conservative estimate of only a 30 years of life expectancy post-lesion acquisition (see Table 8). Significant increases in burden estimates were calculated previously for lymphatic filariasis [24], indicating that mental illness is grossly unaccounted for in the NTD GBD estimates.

These findings come at a crucial time for those affected by CL, a growing number of whom continue to be affected by war and displacement in current conflict zones. The inclusion of iCL into prevalence estimates for CL, we argue, is necessary to enact changes at the policy level that reflect the importance of CL to affected individuals and their communities. Moreover, the studies we have highlighted show a clear benefit for psychological as well as physical therapies on quality of life [41,47,51] as well as rates of depression [41] in CL patients; sadly, inability to access any form of treatment is a commonly cited major concern for patients [45,55,63,64]. As such, there is a very clear opportunity for national NTD programmes and partner international NGOs to incorporate mental health care into their activities and to provide appropriate services to tackle this growing public health problem.

Overall, the stigma and depression linked to NTDs represent areas of global health that have only recently been highlighted [21]. From our literature review, the previous GBD estimates for depression (which predict depressive disorders as a leading cause of DALYs) do not incorporate MDD (or any other mental illness) associated with NTDs. Omitting NTDs from such consideration of global mental health burden is significant as NTDs have been estimated by WHO to affect over 1 billion (1 in 6) people worldwide [1].

### Implications for future GBD studies

In the latest 2016 iteration of the GBD study, the psychological impact of CL scarring has been incorporated into the disease burden estimates for the first time via a modification of disability weights (DW) (IHME personal communication). As such, the disability burden of CL has increased from 41,500 [77] to 273,000 [18] YLDs. Despite this modification, relying upon DWs to capture the unique psychosocial aspects of NTDs has unfortunately led to some of the most stigmatising (namely CL and leprosy) diseases yielding some of the lowest disability (YLD) estimates of all the NTDs in past iterations [18,77–79]. CL is currently viewed as a “*level two disfigurement*”, meaning that its DW reflects “*a visible physical deformity that causes others to stare and comment*. *As a result*, *the person is worried and has trouble sleeping and concentrating*”. This corresponds to a DW of 0.067 in GBD 2016, where 0 indicates perfect health and 1 indicates death [75] Thus, we can be confident that our findings represent an unrecognized mental disease burden of CL.

Instead, we strongly recommend that inactive (scarring) CL be included with active CL infection in future CL prevalence estimates, and that MCL and aCL estimates be presented separately for further information. We have shown that with inactive CL, such a large increase in prevalence (10-fold higher) and burden of co-morbid MDD (8-fold increase) is not sufficiently accounted for by simply altering the DWs for active CL given the evidence of mental illness in patients with residual scarring. As we have only included the “disorder” stage of depressive burden in our YLD estimates, our estimate of CL-related distress (50%) using the staged model approach to depression is not accounted for. Here adjustments to DWs for both aCL and iCL would be justified, as a large proportion of affected individuals with both forms of CL experience some degree of quantifiable distress or socially adverse consequences.

Finally, it is important to highlight that the 2016 GBD Study estimates of aCL incidence [18] are almost half those of previously accepted incidence estimates published in 2012 [71]. This is despite the marked increase in CL incidence due to ongoing conflict and displacement in the Middle East [15]. Similarly, our aCL burden estimates are based upon the 2016 GBD Study estimates of aCL prevalence to allow for comparisons to be made. However, it is unclear why these prevalence estimates are almost seven times lower than the annual incidence of aCL [18] when the majority of cases of aCL self-heal within 6–12 months [2]. For these reasons, we did not include GBD estimates in our calculations of iCL prevalence.

### Study limitations

Although our study is the first to generate prevalence estimates of inactive (scarring) CL, we were cautious of the life span of patients with iCL lesions, which is currently unknown. Whilst the majority of CL infections occur in older children and young adults [4] we took a conservative approach to our iCL prevalence estimates by assuming just 30 years lived with residual scars. Nevertheless, given that the majority of aCL cases occur in the young and working adult populations, this figure could be significantly higher. We also conservatively assume no mortality burden with CL, yet suicidal risk and ideation has been noted in both aCL and iCL patients [34,63].

Secondly, we acknowledge our failure to include prevalence and isolated burden estimates for co-morbid MDD in mucocutaneous leishmaniasis (MCL). As discussed, MCL prevalence (and YLD burden) has not been separated from that of aCL in GBD Studies. A further complicating factor is the mortality rate of MCL, which has not been established and consequently prevented us from generating reliable MCL prevalence estimates from WHO incidence data. Nevertheless, the experience of shame in CL patients [45,59] was surprisingly higher than that found in a study of mixed MCL and CL patients [61]. However, in a study of MCL patients alone [80], notably those with severe disease, rates of social exclusion and reduced quality of life were comparable to those found in CL patients [45,52,54,62]. It is possible that the prevalence of co-morbid MDD in MCL patients is similar to that of aCL patients (~20% of cases), meaning that our aCL burden estimates may be relatively unaffected by the presence of MCL cases within them.

This is the first study to estimate the burden of a co-morbid mental disorder in aCL and iCL. One major limitation of our estimates is the evidence underpinning them. We recognize that our 29 studies represent only a relatively small proportion of the global CL caseload. Nevertheless, our systematic literature review has identified the most evidence of psychological impact in CL patients to date, and doubled the evidence of previous recent attempts [81]. Moreover, these studies represent a range of geographically diverse populations across several levels of economic development. In our analysis, studies quantifying MDD using robust and internationally recognised criteria (i.e. DSM) were given the most weight in generating our final estimates of MDD co-morbid to CL. We were also selective and chose to only utilize studies of CL patients and their care providers. In order to minimize the effects of bias we accounted for patient- and disease-specific variables such as sex, age, lesion location, and country of study. As results for co-morbid MDD were comparable when these variables changed, we were confident that none of these variables could have significantly biased our overall estimates.

Finally, whilst depressive disorders represent the most prevalent form of mental disorder worldwide, CL patients are affected by a range of other mental disorders, which have not been included in our estimates. Indeed, CL patients may be at even greater risk of multiple mental disorders [33]. These include generalised anxiety disorder, which may predominate in the active CL phase [36,38] post-traumatic stress disorder [33] and mixed anxiety and depressive disorder [41], the latter of which is not independently considered within the GBD framework at present.

### Conclusion

Social stigma, disfigurement, and patient suffering are some of the most identifiable features of NTDs, as emphasized by the case of cutaneous leishmaniasis. However, the suffering of those with active infection as well as those who remain disfigured by NTDs post-infection is not adequately factored into NTD programmes or burden estimates. We reason that there is value in striving for both goals by placing the individual at the centre of such programmes to achieve the holistic care of individuals affected by NTDs. After all, focusing on the disease alone ignores the characteristic disability associated with NTDs such as cutaneous leishmaniasis, leprosy, and filariasis, and risks leaving affected individuals behind.

## Supporting information

S1 Appendix Search strategies. (DOCX)

S2 Appendix Inclusion/exclusion criteria. (DOCX)

S3 Appendix Reasons for exclusion following full-text review. (DOCX)

S4 Appendix Summary of CL papers. (DOCX)

S5 Appendix Calculating the prevalence of active and inactive CL. (DOCX)

S6 Appendix Quantifying the burden of co-morbid MDD in cutaneous leishmaniasis. (DOCX)

S7 Appendix PRISMA checklist. (DOCX)
