# Supplementary material for: Cutaneous leishmaniasis and co-morbid major depressive disorder: A systematic review with burden estimates
Source: PLoS Negl Trop Dis. 2019 Feb 25;13(2):e0007092. doi: 10.1371/journal.pntd.0007092 (PMC6405174; doi:10.1371/journal.pntd.0007092)
Supplement: S1 Appendix — (DOCX) [file pntd.0007092.s001.docx]

**S1 Appendix: Search strategies**

**Ovid MEDLINE (R) 1946 to November Week 4 2017^25^**


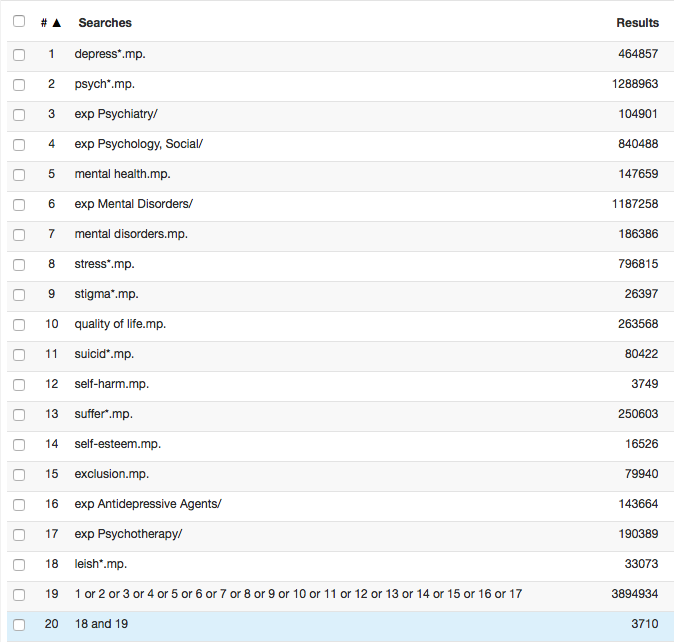


Accessed on 4^th^ December 2017

**Ovid Embase 1974 to 2017 Week 49^26^**


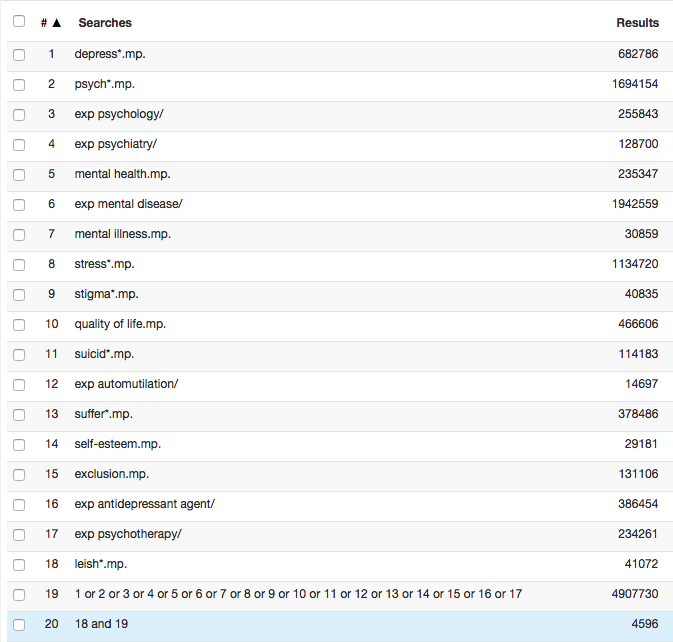


Accessed on 4^th^ December 2017

**Ovid Global Health 1910 to 2017 Week 47^27^**


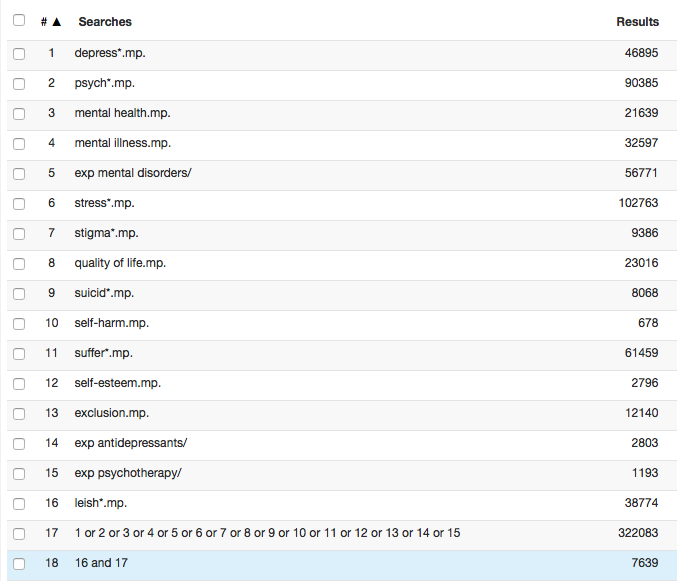


Accessed on 4^th^ December 2017

**PsychINFO 1806 to November Week 2 2017^28^**


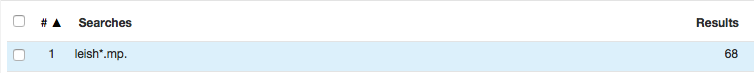


Accessed on 4^th^ December 2017

**LILACS BV Salud^29^**

(tw:((tw:(depres$)) OR (tw:(psych$)) OR (tw:(psico$)) OR (tw:(psiqui$)) OR (tw:(mental health)) OR (tw:(salud mental)) OR (tw:(saúde mental)) OR (tw:(santé mentale)) OR (tw:(enfermedad$ mental$)) OR (tw:(mental disorder$)) OR (tw:(mental disease$)) OR (tw:(stress$)) OR (tw:(stigma$)) OR (tw:(estigma$)) OR (tw:(quality of life)) OR (tw:(qualidade de vida)) OR (tw:(calidad de vida)) OR (tw:(qualité de vie)) OR (tw:(QoL)) OR (tw:(suicid$)) OR (tw:(self-harm)) OR (tw:(suffer$)) OR (tw:(self-esteem)) OR (tw:(exclusion)) OR (tw:(exclusion)) OR (tw:(Antidepressive agents)) OR (tw:(Psychotherapy))))

AND (tw:(leish$))

= 4,828

Accessed on 4^th^ December 2017
