# Supplementary material for: Cutaneous leishmaniasis and co-morbid major depressive disorder: A systematic review with burden estimates
Source: PLoS Negl Trop Dis. 2019 Feb 25;13(2):e0007092. doi: 10.1371/journal.pntd.0007092 (PMC6405174; doi:10.1371/journal.pntd.0007092)
Supplement: S2 Appendix — (DOCX) [file pntd.0007092.s002.docx]

**S2 Appendix: Eligibility criteria**

**Inclusion criteria**

- Primary data presented: MDD/depression diagnosis, depressive symptoms, quality of life, distress, stigma, or other psychosocial or economic concerns
- Qualitative or quantitative studies
- Both active and/or inactive phases of CL
- Both CL and any other form of leishmaniasis present in same study
- English, French, Spanish, Portuguese, and Arabic language studies
- Any period of study until December 4th 2017

**Exclusion criteria**

- Review articles
- Conference abstracts/website articles
- No relevant primary data
- Participants with no previous personal/family experience of CL
- Unclear diagnosis of CL (i.e. inaccurate case definition)
- Sample size <10
