# Supplementary material for: Cutaneous leishmaniasis and co-morbid major depressive disorder: A systematic review with burden estimates
Source: PLoS Negl Trop Dis. 2019 Feb 25;13(2):e0007092. doi: 10.1371/journal.pntd.0007092 (PMC6405174; doi:10.1371/journal.pntd.0007092)
Supplement: S3 Appendix — (DOCX) [file pntd.0007092.s003.docx]

**S3 Appendix: Reasons for exclusion following full-text review**

|  | **Reference** | **Reason** |
| --- | --- | --- |
|  | Adoyo-Adoyo M. (2000) The impact of leishmaniasis in a rural set-up and the effect it has in socio-economic status in the community. Proceedings Sustainable Environmental Management for Poverty Alleviation in the Lake Victoria Basin. 2000; 12-13. | No access |
|  | Ahluwalia IB, Bern C, Costa C, Akter T, Chowdury R, Ali M et al. Visceral leishmaniasis: Consequences of a neglected disease in a Bangladeshi community. Am J Trop Med Hyg. 2003; 69: 624-8. | No relevant primary data |
|  | Akram A, Khan HAA, Qadir A, Sabir AM. A Cross-Sectional Survey of Knowledge, Attitude and Practices Related to Cutaneous Leishmaniasis and Sand Flies in Punjab, Pakistan. PLoS ONE. 2015; 10: e0130929. | No relevant primary data |
|  | Al-Kamel MA. Impact of leishmaniasis in women: a practical review with an update on my ISD-supported initiative to combat leishmaniasis in Yemen (ELYP). Int J Womens Dermatol. 2016; 2: 93-101. | No relevant primary data |
|  | Alemayehu M, Wubshet M, Mesfin N, Gebayehu A. Perceived quality of life among Visceral Leishmaniasis and HIV coinfected migrant male-workers in Northwest Ethiopia: a qualitative study. BMC Public Health. 2017; 17: 204. | CL not included |
|  | Alemayehu M, Wubshet M, Mesfin N, Tamiru A, Gebayehu A. Health-related quality of life of HIV infected adults with and without Visceral Leishmaniasis in Northwest Ethiopia. Health Qual Life Outcomes. 2017; 15: 65. | CL not included |
|  | Alemu A, Alemu A, Esmael N, Dessie Y, Hamdu K, Mathewos B et al. Knowledge, attitude and practices related to visceral leishmaniasis among residents in Addis Zemen town, South Gondar, Northwest Ethiopia. BMC Public Health. 2013; 13: 382. | CL not included |
|  | Al-Ghamdi K, Khurrum H. Successful treatment of atrophic facial leishmaniasis scars by co2 fractional laser. J Cutan Med Surg. 2014; 18: 379-84. | No relevant primary data |
|  | Alonso LM, Alvar J. Stigmatizing neglected tropical diseases: a systematic review. Social Med. 2010; 5: 218-227. | Review article |
|  | Amin TT, Kaliyadan F, Al-Ajyan MI, Al-Arfaj AK, Al-mujhim MA, Al-Harbi SJ et al. Public awareness and attitudes towards cutaneous leishmaniasis in an endemic region in Saudi Arabia. J Eur Acad Dermatol Venereol. 2012; 26: 1544-51. | No relevant primary data |
|  | Arana BA, Rizzo NR, Navin TR, Klein RE, Kroeger A. Cutaneous leishmaniasis in Guatemala: people’s knowledge, concepts and practices. Ann Trop Med Parasitol. 2000; 94: 779-86. | No relevant primary data |
|  | Baghianimoghadam M, Tavakoli B, Ayatollahi K, Mirzaei M. The Effect of education Based on the Theory of Planned Behavior on Preventive Behaviors of Cutaneous Leishmaniasis in Mothers Living in Endemic City of Natanz. J Toloo-e-Behdasht. 2016; 15: 54-66. | No relevant primary data |
|  | Banihashemi M, Nahidi Y, Maleki M, Esmaily H, Moghimi HR. Efficacy of fractional CO2 laser in treatment of atrophic scar of cutaneous leishmaniasis. Lasers Med Sci. 2016; 31: 733-739. | No relevant primary data |
|  | Basher A, Nath P, Nabi SG, Selim S, Rahman MF, Sutradhar SR et al. A Study on Health Seeking Behaviors of Patients of Post-Kala-Azar Dermal Leishmaniasis. BioMed Res Int. 2015; 2015: 314543. | CL not included |
|  | Bellali H, Chemak F, Nouiri I, Ben Mansour D, Ghrab J, Chahed MK. Zoonotic Cutaneous Leishmaniasis Prevalence Among Farmers in Central Tunisia, 2014. J Agromedicine. 2017; 22: 244-250. | No relevant primary data |
|  | Bennis I, De Brouwere V, Belrhiti Z, Sahibi H, Boelaert M. Psychosocial burden of localised cutaneous Leishmaniasis: a scoping review. BMC Public Health. 2018; 18: 358. | Review article |
|  | Boelaert M, Meheus F, Robays J, Lutumba P. Socio-economic aspects of neglected diseases: sleeping sickness and visceral leishmaniasis. Ann Trop Med Parasitol. 2010; 104: 535-42. | Review article |
|  | Borges BKA, da Silva JA, Haddad JPA, Moreira EC, de Magalhães DF, Ribeiro LML et al. Avaliação do nível de conhecimento e de atitudes preventivas da população sobre a leishmaniose visceral em Belo Horizonte, Minas Gerais, Brasil. Cad Saúde Pública. 2008; 24: 777-784. | CL not included |
|  | Bouratbine A, Moussa H, Aoun K, Benismail R. Anthropologic research and understanding pediatric visceral leishmaniasis in Tunisia. Bull Soc Pathol Exot. 1998; 91: 183-7. | CL not included |
|  | Camargo LB, Langoni H. Impact of leishmaniasis on public health. J Venom Anim Toxins. 2006; 12: 527-548. | No relevant primary data |
|  | Camino L & Anderson A. Lenguaje, conocimiento y mito: análisis del saber empírico sobre Leishmaniasis en minorías étnicas y grupos mestizos. Rev Peru Epidemiol. 1992; 5: 32-35. | No relevant primary data |
|  | Carmo RF, da Luz ZMP, Bevilacqua PD. Percepções da população e de profissionais de saúde sobre a leishmaniose visceral. Ciênc saúde coletiva. 2016; 21: 621-628. | CL not included |
|  | Carrillo-Bonilla LM, Trujillo JJ, Alvarez-Salas L, Vélez-Bernal ID. Study of knowledge, attitudes, and practices related to leishmaniasis: evidence of government neglect in the Colombian darien. Cad Saude Publica. 2014; 30: 2134-44. | No relevant primary data |
|  | Casavechia MTG, de Araújo SM, Teixeira JJV, Lonardoni MVC. A leishmaniose tegumentar sob a perspectiva do paciente: resultado de uma prática educativa. Rev Bras Anal Clin. 2002; 34: 233-239. | No access |
|  | Cavalcante LL. Processo de trabalho da ESF Vida e Esperança do município de São João do Pacuí para o acompanhamento de vítimas de leishmaniose tegumentar american. Ph.D. Thesis, Universidade Federal de Minas Gerais. 2015. Available from:  http://www.bibliotecadigital.ufmg.br/dspace/bitstream/handle/1843/BUOS-ANPJQP/tcc_lucas_leite_cavalcante.pdf?sequence=1. | No relevant primary data |
|  | Costa JM, Vale KC, Cecílio IN, Osaki NK, Netto EM, Tada MS et al. Aspectos psicossociais e estigmatizantes da leishmaniose cutâneo-mucosa. Rev Soc Brasil Med Trop. 1987; 20: 77-82. | CL not included |
|  | Costa KLFdL. Percepção e diagnóstico da Leishmaniose visceral canina em áreas ribeirinhas na cidade de Mossoró, Rio Grande do Norte. M.Sc. Thesis, Universidade Federal Rural do Semi-Árido. 2014. Available from: https://ppgats.ufersa.edu.br/wp-content/uploads/sites/47/2014/09/Dissertação-final-Kalidia-Felipe.pd. | CL not included |
|  | da Silva MRB. Percepção do corpo pela mulher com leishmaniose tegumentar americana: uma análise compreensiva. M.Sc. Thesis, Universidade Federal da Bahia. 2001. | No access |
|  | Dedet JP, Pillot B, Gentilini M. Evaluation of the socioeconomic costs of cutaneous leishmaniasis in French Guiana. Rev Epidemiol Sante Publique. 1991; 39: 129-33. | No relevant primary data |
|  | de Magalhaes DF, da Silva JA, Haddad JP, Moreira EC, Fonseca MI, de Ornelas ML et al. Dissemination of information on visceral leishmaniasis from schoolchildren to their families: a sustainable model for controlling the disease. Cad Saude Publica. 2009; 25: 1642-6. | CL not included |
|  | de Moura LMA, do Monte NDP, de Sousa RLT, dos Santos JP, Freire SM. Nível dos Conhecimentos Sobre as Leishmanioses Visceral e Tegumentar pela População de Alguns Bairros de Teresina, Piauí, Brasil. UNOPAR Cient Ciênc Biol Saúde. 2015; 17: 21-30. | No relevant primary data |
|  | Dobles-Ulloa A, Perriard C. Representations, attitudes and practices related to cutaneous leishmaniasis in people from Acosta County, San Jose Province, Costa Rica. An exploratory anthropological study. Cad Saude Publica. 1994; 10: 181-9. | No relevant primary data |
|  | dos Reis DC, Gazzinelli A, Silva CAdB, Gazzinelli MF. Health education and social representation: an experience with the control of tegumentary leishmaniasis in an endemic area in Minas Gerais, Brazil. Cad. Saúde Pública. 2006; 22: 2301-2310. | No relevant primary data |
|  | Ducrotoy MJ, Yahyaoui Azami H, El Berbri I, Bouslikhane M, Fassi Fihri O, Boué F et al. Integrated health messaging for multiple neglected zoonoses: Approaches, challenges and opportunities in Morocco. Acta Trop. 2015; 152: 17-25. | No relevant primary data |
|  | Fincher CL, Thornhill R, Murray DR, Schaller M. Pathogen prevalence predicts human cross-cultural variability in individualism/collectivism. Proc Biol Sci. 2008; 275: 1279-85. | No relevant primary data |
|  | Gama MEA, Barbosa JdS, Pires B, Cunha AKB, Freitas AR, Ribeiro IR et al. Evaluation of the level of knowledge of the local population about visceral leishmaniasis in endemics areas of Maranhão State, Brazil. Cad. Saúde Pública. 1998; 14: 381-390. | CL not included |
|  | Gerstl S, Amsalu R, Ritmeijer K. Accessibility of diagnostic and treatment centres for visceral leishmaniasis in Gedaref State, northern Sudan. Trop Med Int Health. 2006; 11: 167-175. | CL not included |
|  | González U, Pinart M, Rengifo-Pardo M, Macaya A, Alvar J, Tweed JA. Interventions for American cutaneous and mucocutaneous leishmaniasis. Cochrane Database Syst Rev. 2009; CD004834. | Review article |
|  | Gooneratne BWM. A remarkable description of 'Aleppo boil' in 1839. Trans R Soc Trop Med Hyg. 1970; 64: 197. | Small sample size |
|  | Gouveia C. Leishmaniose tegumentar americana no caminho da Cachoeira, Colônia Juliano Moreira, Campus Fiocruz da Mata Atlântica, Jacarepaguá, Rio de Janeiro: indicadores entomológicos e educação popular em saúde. Ph.D. Thesis, Escola Nacional de Saúde Pública Sergio Arouca. 2006. | No access |
|  | Gouveia C, de Oliveira RM, Zwetsch A, Motta-Silva D, Carvalho BM, de Santana AF et al. Integrated tools for American cutaneous leishmaniasis surveillance and control: intervention in an endemic area in Rio de Janeiro, RJ, Brazil. Interdiscip Perspect Infect Dis. 2012; 2012: 568312. | No relevant primary data |
|  | Guevara BC. Aporte de la etnografía en el conocimiento de los códigos socioculturales de la leishmaniasis cutánea localizada en un programa de educación para la salud, en Venezuela. Cad Saúde Pública Rio de Janeiro. 2007. 23: S75-S83. | No relevant primary data |
|  | Guthmann JP, Calmet J, Rosales E, Cruz M, Chang J, Dedet JP. Patients' associations and the control of leishmaniasis in Peru. Bull World Health Org. 1997; 75: 39-44. | No relevant primary data |
|  | Hofstraat K, van Brakel WH. Social stigma towards neglected tropical diseases: a systematic review. Int Health. 2016; 8: i53-70. | Review article |
|  | Homsi Y, Makdisi G. Leishmaniasis: A forgotten disease among neglected people. Internet J Health. 2009; 11. | No relevant primary data |
|  | Isaza DM, Restrepo BN, Arboleda M, Casas E, Hinestroza H, Yurgaqui T. La leishmaniasis: conocimientos y prácticas en poblaciones de la costa del Pacífico de Colombia, Rev Panam Salud Publica/Pan Am J Public Health. 1999; 6: 177-184. | No relevant primary data |
|  | Jaffary F, Nilforoushzadeh MA, Siadat A, Haftbaradaran E, Ansari N, Ahmadi E. A Comparison between the Effects of Glucantime, Topical Trichloroacetic Acid 50% plus Glucantime, and Fractional Carbon Dioxide Laser plus Glucantime on Cutaneous Leishmaniasis Lesions. Dermatol Res Pract. 2016; 2016: 6462804. | No relevant primary data |
|  | Jahan S, Al-Saigul AM, Nimir SE, Mustafa AS. Priorities for primary health care research in Qassim, central Saudi Arabia. Saudi Med J. 2014; 35: 298-303. | No relevant primary data |
|  | Kaplanski G, Deharo C, Koeppel MC, Durand JM, David M, Farnarier C et al. Cutaneo-visceral leishmaniasis after a severe nervous depression, Rev Med Interne. 1995; 16: 55-7. | Small sample size |
|  | Karimkhani C, Wanga V, Coffeng LE, Naghavi P, Dellavalle RP, Naghavi M. Global burden of cutaneous leishmaniasis: a cross-sectional analysis from the Global Burden of Disease Study 2013. Lancet Infect Dis. 2016; 16: 584-591. | No relevant primary data |
|  | Kashfi SM, Khani Jeihooni A, Rezaeianzade A. Effect of Health Workers’ Training Programs on Preventive Behavior of Leishmaniosis Based on BASNEF Model. J Res Health Sci. 2012; 12: 114-8. | No relevant primary data |
|  | Kasili S, Okindo ES, Kutima HL, Mutai JM. Socioeconomic Impacts of Leishmaniases on Households of Marigat Sub County, Baringo County of Kenya. J Trop Dis. 2016; 4: 226. | CL not included |
|  | Kassi M, Kassi M, Afghan AK, Rehman R, Kasi PM. Marring leishmaniasis: the stigmatization and the impact of cutaneous leishmaniasis in Pakistan and Afghanistan. PLoS Negl Trop Dis. 2008; 2: e259. | No relevant primary data |
|  | Kebede N, Worku A, Ali A, Animut A, Negash Y, Gebreyes WA et al. Community knowledge, attitude and practice towards cutaneous leishmaniasis endemic area Ochello, Gamo Gofa Zone, South Ethiopia. Asian Pac J Trop Biomed. 2016; 6: 562-567. | No relevant primary data |
|  | Khatami A, Firooz A, Gorouhi F, Dowlati Y, Treatment of acute Old World cutaneous leishmaniasis: A systematic review of the randomized controlled trials, J Am Acad Dermatol. 2007 Aug;57(2):335.e1-29. | Review article |
|  | Koirala S, Parija SC, Karki P, Das ML. Knowledge, attitudes, and practices about kala-azar and its sandfly vector in rural communities of Nepal. Bull World Health Organ. 1998; 76: 485-90. | CL not included |
|  | Kumar N, Siddiqui NA, Verma RB, Das P. Knowledge about sandflies in relation to public and domestic control activities of kala-azar in rural endemic areas of Bihar. J Commun Dis. 2009; 41: 121-8. | No access |
|  | Kumar N, Siddiqui NA, Verma R, Kar SK. Women's perceptions on kala-azar. J Parasit Dis. 2002; 26: 38-41. | No access |
|  | Layegh P, Teimourian M, Shiva F, Hebrani P, Momenzadeh A. Quality of life, anxiety and depression in children with cutaneous leishmaniasis: Major concerns or not? Pediatr Dermatol. 2017; 34: P-122 | Conference abstract |
|  | Li F, Yang J-K, Liu L-L, Yang S-J, Gansu Provincial Center for Disease Control and Prevention. Investigation on current situation of correct knowledge about prevention and treatment for kala-azar in Wen County, Gansu Province. Bull Dis Control Prevent. 2013. | No access |
|  | Litt E, Baker MC, Molyneux D. Neglected tropical diseases and mental health: a perspective on comorbidity. Trends Parasitol. 2012; 28: 195-201. | Review article |
|  | Lolli MCGdS, Lolli LF, Gualda KP, Silva LL, Marcon SS, Pelloso SM et al. Observations about epidemiology and awareness of American tegumentary leishmaniasis in endemic region in southern Brazil, Biosci J Uberlândia. 2011; 27: 849-855. | No relevant primary data |
|  | López K, Tartaglino LC, Steinhorst II, Santini MS, Salomón OD. Factores de riesgo, representaciones y prácticas asociadas con la leishmaniasis visceral humana en un foco urbano emergente en Posadas, Argentina. Biomédica. 2016; 36: 51-63. | CL not included |
|  | López-Perea N, Sordo L, Gadisa E, Cruz I, Hailu T, Moreno J et al. Knowledge, Attitudes and Practices Related to Visceral Leishmaniasis in Rural Communities of Amhara State: A Longitudinal Study in Northwest Ethiopia. PLoS Negl Trop Dis. 2014; 8: e2799. | CL not included |
|  | Magalhães HM, Costa JML, Costa RM, França F, Vale KC, Marsden P et al. Mudança do componente cognitivo da atitude de uma população de região endêmica do Sul da Bahia diante da leishmaniose tegumentar. Rev Soc Bras Med Trop. 1990; 23: 49-52. | No relevant primary data |
|  | Maia CS, Pimentel DS, Santana MA, Oliveira GM, Faustino MAG, Alves LC. The perception of the risk factors associated with American Visceral Leishmaniasis in Petrolina, Pernambuco, Brazil. Med Veterinária Recife. 2013; 7: 19-25. | CL not included |
|  | Mans DRA, Zeegelaar JE, Hu RVPF, Kent AD, Ramdas S, Schallig HDFH. Clinical, biological, and anthropological aspects of leishmaniasis in Suriname - report of the final meeting of the Integrated Program. Academic J Suriname. 2014; 5: 464-473. | No relevant primary data |
|  | Marrero C, Fernández D, González C. Plan to establish an information campaign about insect vectors of disease at Rio Anus, a rural community near Guanare (Portuguesa state, Venezuela). Rev Unell Cienc Tec. 2006; 24: 17-26. | No relevant primary data |
|  | Martins ACdC, Nunes JA, Paheco SJB, de Souza CTV. Percepção do risco de transmissão de zoonoses em um Centro de Referência. Rev Eletron de Comun Inf Inov Saúde. 2015. 9. | No relevant primary data |
|  | Mishra RN, Singh SP, Vanlerberghe V, Sundar S, Boelaert M, Lefèvre P. Lay perceptions of kala-azar, mosquitoes and bed nets in Bihar, India. Trop Med Int Health. 2010; 15: 36-41. | CL not included |
|  | Moreira RsCR, Rebelo JMM, Gama MEA, Costa JML. Nível de conhecimentos sobre Leishmaniose Tegumentar Americana (LTA) e uso de terapias alternativas por populações de uma área endêmica da Amazônia do Maranhão, Brasil. Cad Saúde Pública. 2002; 18: 187-195. | No relevant primary data |
|  | Nandha B, Srinivasan R, Jambulingam P. Cutaneous leishmaniasis: knowledge, attitude and practices of the inhabitants of the Kani forest tribal settlements of Tiruvananthapuram district, Kerala, India. Health Educ Res. 2014; 29: 1049-57. | No relevant primary data |
|  | Netto EM, Tada MS, Golightly L, Kalter DC, lago E, Barreto AC et al. Conceitos de uma população local a respeito da leishmaniose mucocutânea em uma área endêmica. Rev Soc Bras Med Trop. 1985; 18: 33-37. | CL not included |
|  | Nieves E, Villarreal N, Rondón M, Sánchez M, Carrero J. Evaluation of knowledge and practice on tegumentary leishmaniasis in an endemic area of Venezuela. Biomédica. 2008; 28: 347-356. | No relevant primary data |
|  | Nikookar SH, Pashaeei T, Nikzad D, Moosa-Kazemi SH, Davari B. Assessment of Knowledge, Attitude and Practice toward Vectors Control Programs among Managers of the Schools in Neka, Iran. Iran J Health Sci. 2015; 3: 58-62. | No relevant primary data |
|  | Nilforoushzadeh MA, Jaffary F, Ansari N, Moradi S, Siadat AH. The comparison between trichloroacetic Acid 50% and co(2) laser in the treatment of cutaneous leishmaniasis scar. Indian J Dermatol. 2011; 56: 171-3. | No relevant primary data |
|  | Nilforoushzadeh MA, Minaravesh S, Jaffary F, Siadat AH, Haftbaradaran E. Comparison the efficacy of ablative CO2 laser and fractional CO2 laser on the healing of cutaneous leishmaniasis scars. Adv Biomed Res. 2014; 3. | No relevant primary data |
|  | Nilforoushzadeh MA, Esfahani MH, Fesharaki MA, Siadat AH, Ansari N, Baradaran EH. Treatment of atrophic cutaneous leishmaniasis scar using autologous fibroblasts and keratinocytes (a case report and literature review). J Res Med Sci. 2010; 15: 125-6. | No relevant primary data |
|  | Odonne G, Berger F, Stien D, Grenand P, Bourdy G. Treatment of leishmaniasis in the Oyapock basin (French Guiana): A K.A.P. survey and analysis of the evolution of phytotherapy knowledge amongst Wayãpi Indians. J Ethnopharmacol. 2011; 137: 1228-39. | No relevant primary data |
|  | Odonne G, Bourdy G, Castillo D, Estevez Y, Lancha-Tangoa A, Alban-Castillo J et al. Ta’ta’, Huayani: Perception of leishmaniasis and evaluation of medicinal plants used by the Chayahuita in Peru. Part II. J Ethnopharmacol. 2009; 126: 149-58. | No relevant primary data |
|  | Okindo EG, Kutima HL, Mutai J, Kasili S. Attitude and practices of household heads towards leishmaniases infections in Marigat sub-county, Baringo County, Kenya. East African Med J. 2017; 94: 86-94. | No access |
|  | Okwa OO. Tropical parasitic diseases and women. Ann Afr Med. 2007; 6: 157-163. | Review article |
|  | Okwor I, Uzonna J. Social and economic burden of human leishmaniasis. Am J Trop Med Hyg. 2016; 94: 489-93. | Review article |
|  | Osman AM, Almuslet NA. Evaluation of CO2 laser efficacy in the treatment of cutaneous leishmaniasis in a group of 10 Sudanese patients. Photonics Lasers Med. 2015; 4: 259-263. | No access |
|  | Osman Y, Wayit S, Zhu CZ, Tong SX, Wu WP, Jumahun R et al. Change of Knowledge Rate after Health Education on Visceral Leishmaniasis among Residents in a Township of Kashgar City. Zhongguo Ji Sheng Chong Xue Yu Ji Sheng Chong Bing Za Zhi. 2008; 26: 236-241. | CL not included |
|  | Ozaki M, Islam S, Rahman KM, Rahman A, Luby SP, Bern C. Economic consequences of post-kala-azar dermal leishmaniasis in a rural Bangladeshi community. Am J Trop Med Hyg. 2011; 85: 528-534. | CL not included |
|  | Pal B, Murti K, Siddiqui NA, Das P, Lal CS, Babu R et al. Assessment of quality of life in patients with post kalaazar dermal leishmaniasis. Health Qual Life Outcomes. 2017; 15: 148. | CL not included |
|  | Pardo RH, Carvajal A, Ferro C, Davies CR. Effect of knowledge and economic status on sandfly control activities by householders at risk of cutaneous leishmaniasis in the subandean region of Huila department, Colombia. Biomedica. 2006; 26: 167-79. | No relevant primary data |
|  | Patiño-Londoño SY, Salazar LM, Acero CT, Bernal IDV. Aspectos socioepidemiológicos y culturales de la leishmaniasis cutánea: concepciones, actitudes y prácticas en las poblaciones de Tierralta y Valencia, (Córdoba, Colombia). Salud Colectiva. 2017; 13: 123-138. | No relevant primary data |
|  | Pimenta DN, Leandro A, Schall VT. Aesthetics of the grotesque and audiovisual production for health education: segregation or empathy? The case of leishmaniasis in Brazil. Cad Saúde Pública Rio de Janeiro. 2007; 23: 1161-1171. | No relevant primary data |
|  | Rakhshani T, Seyyed MK, Ebrahimi MR, Taravatmanesh S, Rasheki M. Knowledge, Attitude and Practice of the Households about Prevention of Cutaneous Leishmaniasis, Iran, Shiraz at 2016. Environ Health Promot. 2017; 2: 186-192. | No relevant primary data |
|  | Ramdas S. Perceptions and treatment of cutaneous leishmaniasis in Suriname: a medical-anthropological perspective. Ph.D. Thesis, Amsterdam Institute for Social Science Research. 2015. Available from: https://pure.uva.nl/ws/files/2432751/154891_Ramdas_thesis_met_cover.pdf. | Repeated in subsequent publication |
|  | Ramdas S. Cruel disease, cruel medicine: self-treatment of cutaneous leishmaniasis with harmful chemical substances in Suriname. Soc Sci Med. 2012; 75: 1097-105. | No relevant primary data |
|  | Rangel JAC, Moreno REC. Leishmaniasis cutánea: epidemiología y aspectos psicosociales. Ph.D. Thesis, Universidad Central de Venezuela. 2008. | No access |
|  | Reis ACP. Barra do Corumbê, Paraty-RJ: Leishmaniose e representações sociais. Ph.D. Thesis, Escola Nacional de Saúde Pública. 2004. | No access |
|  | Reveiz L, Maia-Elkhoury ANS, Nicholls RS, Sierra Romero GA, Yadon ZE. Interventions for American Cutaneous and Mucocutaneous Leishmaniasis: A Systematic Review Update. PLoS ONE. 2013; 8: e61843. | Review article |
|  | Reyburn H, Rowland M, Mohsen M, Khan B, Davies C. The prolonged epidemic of anthroponotic cutaneous leishmaniasis in Kabul, Afghanistan: 'Bringing down the neighbourhood'. Trans R Soc Trop Med Hyg. 2003; 97: 170-176. | No access |
|  | Rios-González C, Flores J, Calvopiña M. Conocimientos sobre Leishmania spp. y leishmaniasis en estudiantes de medicina de Latinoamérica. Rev Chil Infectol. 2016; 33: 481-482. | No relevant primary data |
|  | Saberi S, Zamani A, Motamedi N, Nilforoushzadeh MA, Jaffary F, Rahimi E et al. The knowledge, attitude, and prevention practices of students regarding cutaneous leishmaniasis in the hyperendemic region of the Shahid Babaie Airbase. Vector Borne Zoonotic Dis. 2012; 12: 306-9. | No relevant primary data |
|  | Sada-e-azadi. Leishmaniasis causes social stigma. 20 May 2011. Available from: http://www.sada-e-azadi.net/index.php/en/topic/46-heath/4243-leishmaniasis-causes-social-stigma. Cited 15 March 2018. | Website article |
|  | Sangueza OP, Sangueza JM, Stiller MJ, Sangueza P. Mucocutaneous leishmaniasis: A clinicopathologic classification. J Am Acad Dermatol. 1993; 28: 927-32. | No relevant primary data |
|  | Santos JB, Lauand L, de Souza GS, Macêdo VdO. Fatores sócio-econômicos e atitudes em relação à prevenção domiciliar da leishmaniose tegumentar americana, em uma área endêmica do sul da Bahia, Brasil. Cad Saúde Pública Rio de Janeiro. 2000; 16: 701-708. | No relevant primary data |
|  | Sarkari B, Qasem A, Shafaf MR. Knowledge, attitude, and practices related to cutaneous leishmaniasis in an endemic focus of cutaneous leishmaniasis, Southern Iran. Asian Pac J Trop Biomed. 2014; 4: 566-9. | No relevant primary data |
|  | - Schallig H, Kent A, Ramdas S, Hu R, Mans D, van der Geest S et al. Leishmaniasis in suriname-new insights into a neglected disease. Am Soc Trop Med Hyg. 2014; 91: 333. | Conference abstract |
|  | Scorza JV, Rojas E. La leishmaniasis tegumentaria venezolana: problemática contemporánea en el Estado Trujillo. Bol Dir Malariol Saneam Ambient. 1990; 30: 1-6. | No access |
|  | Siddiqui NA, Kumar N, Ranjan A, Pandey K, Das VN, Verma RB et al. Awareness about kala-azar disease and related preventive attitudes and practices in a highly endemic rural area of India. Southeast Asian J Trop Med Public Health. 2010; 41: 1-12. | No access |
|  | Singh SP, Reddy DC, Mishra RN, Sundar S. Knowledge, attitude, and practices related to Kala-azar in a rural area of Bihar State, India. Am J Trop Med Hyg. 2006; 75: 505-8. | CL not included |
|  | Srinivasan R, Ahmad T, Raghavan V, Kaushik M, Pathak R. Positive Influence of Behavior Change Communication on Knowledge, Attitudes, and Practices for Visceral Leishmaniasis/Kala-azar in India. Glob Health Sci Pract. 2018; 6: 192-209. | CL not included |
|  | Stewart CC, Brieger WR. Community views on Cutaneous Leishmaniasis in Istalif, Afghanistan: implications for treatment and prevention. Int Q Community Health Educ. 2008; 29: 123-42. | No relevant primary data |
|  | Strømme EM, Baerøe K, Norheim OF. Disease control priorities for neglected tropical diseases: lessons from priority ranking based on the quality of evidence, cost effectiveness, severity of disease, catastrophic health expenditures, and loss of productivity. Dev World Bioeth. 2014; 14: 132-41. | No relevant primary data |
|  | Sunyoto T, Atia AM, Khalid G, Alcoba G, den Boer M, Boelaert M. Suffering in silence: A qualitative study on reasons of delay in seeking care for visceral leishmaniasis in southern Gedaref, Sudan. Trop Med Int Health. 2017; 22: 67-68. | No access |
|  | Sunyoto T, Potet J, Picado A, Boelaert M. Evaluating access barriers to visceral Leishmaniasis diagnostic and drugs in 6 endemic countries in eastern Africa: Is regional approach the solution? Trop Med Int Health. 2017; 22: 265. | No access |
|  | Thornton SJ, Wasan KM, Piecuch A, Lynd LL, Wasan EK. Barriers to treatment for visceral leishmaniasis in hyperendemic areas: India, Bangladesh, Nepal, Brazil and Sudan. Drug Dev Ind Pharm. 2010; 36: 1312-9. | CL not included |
|  | Uchôa CMA, Serra CMB, Magalhães CdM, da Silva III RMM, Figliuolo LP, Leal CA et al. Educação em saúde: ensinando sobre a leishmaniose tegumentar americana. Cad Saúde Pública Rio de Janeiro. 2004; 20: 935-941. | No relevant primary data |
|  | Vahabi A, Rassi Y, Oshaghi M, Vahabi B, Rafizadeh S, Sayyad S et al. First survey on Knowledge, Attitude and Practice about Cutaneous Leishmaniasis among dwellers of Musian district, Dehloran County, Southwestern of Iran, 2011. survey on Knowledge, Attitude and Practice about Cutaneous Leishmaniasis among dwellers of Musian district, Dehloran County, Southwestern of Iran. Life Sci J. 2013; 1010: 864-868. | No relevant primary data |
|  | van 't Noordende AT, Kuiper H, Ramos AN Jr, Mieras LF, Barbosa JC, Pessoa SM et al. Towards a toolkit for cross-neglected tropical disease morbidity and disability assessment. Int Health. 2016; 8: i71-81. | CL not included |
|  | Vázquez ML, Kroeger A, Lipowsky R, Alzate A. Conceptos populares sobre la leishmaniasis cutánea en Colombia y su aplicabilidad en programas de control. Bol Oficina Sanit Panam. 1991; 110: 402-415. | No relevant primary data |
|  | Weigel MM, Armijos RX. The traditional and conventional medical treatment of cutaneous leishmaniasis in rural Ecuador. Rev Panam Salud Publica. 2001; 10: 395-404. | No relevant primary data |
|  | Wilsher EJ. The impact of Neglected Tropical Diseases, and their associated stigma, on people's basic capabilities. M.Sc. Thesis, Durham University. 2011. Available from:  http://etheses.dur.ac.uk/3301/1/THESIS_FINALpdf.pdf?DDD14+. | Review article |
|  | Blas E, Kurup AS, World Health Organization. Equity, social determinants and public health programmes. Geneva : World Health Organization. 2010. | No relevant primary data |
|  | World Health Organization. Neglected disease, lasting stigma – Leishmaniasis continues to affect the lives of tens of thousands of Afghans. Jan 22 2017. Available from: http://www.emro.who.int/afg/afghanistan-news/leishmaniasis-neglected-disease.html. Cited 15 March 2018. | Website article |
|  | Yared S, Deribe K, Gebreselassie A, Lemma W, Akililu E, Kirstein OD et al. Risk factors of visceral leishmaniasis: a case control study in north-western Ethiopia. Parasit Vectors. 2014; 14: 470. | CL not included |

| **Reason** | **Number** |
| --- | --- |
| No relevant primary data | 61 |
| CL not included | 27 |
| No access | 16 |
| Review article | 11 |
| Conference abstract | 2 |
| Website article | 2 |
| Small sample size | 2 |
| Repeated in subsequent publication | 1 |
| **Total** | **122** |
