# Supplementary material for: Cutaneous leishmaniasis and co-morbid major depressive disorder: A systematic review with burden estimates
Source: PLoS Negl Trop Dis. 2019 Feb 25;13(2):e0007092. doi: 10.1371/journal.pntd.0007092 (PMC6405174; doi:10.1371/journal.pntd.0007092)
Supplement: S4 Appendix — (DOCX) [file pntd.0007092.s004.docx]

**S4 Appendix: Summary of CL papers**

| **Reference** | **Country**  **(WHO Region)** | **MDD Diagnosis** | **MDD Symptoms** | **Quality of Life** | **Psychological Distress** | **Stigma, Disfigurement,**  **& Socio-economic** | **Qualitative** |
| --- | --- | --- | --- | --- | --- | --- | --- |
| Simsek *et al*  (2008)^33^ | Turkey  (EURO)) |  |  |  |  |  |  |
| Torkashvand *et al*  (2016)^34^ | Iran  (EMRO) |  |  |  |  |  |  |
| Turan *et al*  (2015)^36^ | Turkey  (EURO) |  |  |  |  |  |  |
| Yanik *et al*  (2004)^38^ | Turkey  (EURO) |  |  |  |  |  |  |
| Honório *et al*  (2016)^40^ | Brazil  (AMRO) |  |  |  |  |  |  |
| Hu *et al*  (2015)^41^ | Suriname  (AMRO) |  |  |  |  |  |  |
| Al-Kamel  (2017)^42^ | Yemen  (EMRO) |  |  |  |  |  |  |
| Bastidas *et al*  (2008)^43^ | Venezuela  (AMRO) |  |  |  |  |  |  |
| Pacheco *et al*  (2017)^44^ | Brazil  (AMRO) |  |  |  |  |  |  |
| Semeneh  (2012)^45^ | Ethiopia  (AFRO) |  |  |  |  |  |  |
| Chahed *et al*  (2016)^46^ | Tunisia  (EMRO) |  |  |  |  |  |  |
| Elsaie *et al*  (2017)^47^ | Egypt  (EMRO) |  |  |  |  |  |  |
| Handjani *et al*  (2013)^49^ | Iran  (EMRO) |  |  |  |  |  |  |
| Nilforoushzadeh *et al*  (2009)^51^ | Iran  (EMRO) |  |  |  |  |  |  |
| Ranawaka *et al*  (2014)^52^ | Sri Lanka  (SEARO) |  |  |  |  |  |  |
| Toldeo Jr *et al*  (2013)^53^ | Brazil  (AMRO) |  |  |  |  |  |  |
| Vares *et al*  (2013)^54^ | Iran  (EMRO) |  |  |  |  |  |  |
| Bennis *et al*  (2017)^55^ | Morocco  (EMRO) |  |  |  |  |  |  |
| Abazid *et al*  (2012)^57^ | Syria  (EMRO) |  |  |  |  |  |  |
| Fernando *et al*  (2010)^58^ | Sri Lanka  (SEARO) |  |  |  |  |  |  |
| Ramdas *et al*  (2016)^59^ | Suriname  (AMRO) |  |  |  |  |  |  |
| Reithinger *et al*  (2005)^60^ | Afghanistan  (EMRO) |  |  |  |  |  |  |
| Ruoti *et al*  (2013)^61^ | Paraguay  (AMRO) |  |  |  |  |  |  |
| Weigel *et al*  (1994)^62^ | Ecuador  (AMRO) |  |  |  |  |  |  |
| Alorfi  (2016)^63^ | Saudi Arabia  (EMRO) |  |  |  |  |  |  |
| da Silva *et al*  (2004)^64^ | Brazil  (AMRO) |  |  |  |  |  |  |
| Guevara  (2007)^65^ | Venezuela (AMRO) |  |  |  |  |  |  |
| Martins  (2014)^66^ | Brazil  (AMRO) |  |  |  |  |  |  |
| Reyburn *et al*  (2000)^67^ | Afghanistan  (EMRO) |  |  |  |  |  |  |
