# Supplementary material for: Cutaneous leishmaniasis and co-morbid major depressive disorder: A systematic review with burden estimates
Source: PLoS Negl Trop Dis. 2019 Feb 25;13(2):e0007092. doi: 10.1371/journal.pntd.0007092 (PMC6405174; doi:10.1371/journal.pntd.0007092)
Supplement: S5 Appendix — (DOCX) [file pntd.0007092.s005.docx]

**S5 Appendix: Calculating the prevalence of active and inactive CL**

**Background:**

- The prevalence of the forms of cutaneous leishmaniasis ranging from parasitologically active to inactive

CL lesions to the variable presence of MCL lesions forms a spectrum of disease^3^

- Active CL heals to inactive CL in a variable amount of time, typically in ~1 year^2^
- “CL” prevalence data refers to active CL and does not include inactive CL
- “CL” prevalence data also includes MCL cases in both WHO data and GBD studies

**Data Sources:**

- Based upon GHO reported incidence data for 2006-2015 for active CL (MCL included)^70^ along with previously published global prevalence estimates for active CL^10,71^
- To remove cases of MCL from CL data, a fixed rate was calculated from WHO technical documents^72-74^
- It should be noted that the GBD published data was not used to inform our iCL prevalence estimates for the following reasons:
- The most recent GBD incidence figures for 2016^18^ are around half those of the previously estimated by Alvar *et al* (2012)^71^ when the incidence of CL has been increasing;
- The most recent GBD prevalence data for aCL was 7 times higher than its incidence,^18^ yet the mean duration of aCL is ~1 year^2^ and iCL is not included in these prevalence figures

| **Reported and estimated incidence and prevalence of cutaneous leishmaniasis, 2002-2016 (*MCL included)** | | | | | |
| --- | --- | --- | --- | --- | --- |
| **Author** | **Study Year** | **Reported** | | **Estimated** | |
|  |  | **Incidence** | **Prevalence** | **Incidence** | **Prevalence** |
| Mathers *et al*^10^ | 2002 | - | - | 1,157,000 | 2,157,000 |
| Alvar *et al*^71^ | 2002-2009 | 214,036 | - | 1,213,300 | - |
| WHO GHO^70^ | 2006-2015 | 188,244  (mean)  163,078 (min)  211,646 (max) | 1,882,438  (10 years) | - | - |
| GBD 2010^79^ | 2010 | - | - | - | 10,000,000* |
| GBD 2013^78^ | 2013 | - | - | - | 3,914,800* |
| GBD 2015^77^ | 2015 | - | - | - | 3,895,900* |
| GBD 2016^18^ | 2016 | - | - | 678,000* | 4,320,000* |

N.B. The studies below the dotted line ( **- - -** ) refer to Global Burden of Disease (GBD) studies conducted by the Institute of Health Metrics and Evaluation (IHME)

**Methodology:**

- Overall, 5-7x difference between reported and estimates incidence of CL (with MCL removed);^10,70,71^
- 30 years life expectancy with aCL/iCL conservatively estimated
- As such, the reported 10-year incidence from WHO was multiplied by 6 (representing 10 years

estimated prevalence), and further multiplied by 3 (to reflect 30 years of life expectancy with inactive CL).

Based upon our calculations and assumptions listed below, the prevalence of this spectrum of disease exists in the following ratio:

|  | **Active CL**  (GBD 2016)^18^ | **Inactive CL** | **Total** |
| --- | --- | --- | --- |
| **Ratio** | ~10 | ~90 | 100 |
| **Prevalence** | 4,320,000 | 33,883,900 | 38,203,900 |

**Assumptions:**

- Negligible mortality for CL (despite suicidal risk and ideation being noted in both iCL^34^ and aCL^63^ patients)

• Impact of treatment (treated patients are at lower risk of objective severity of disease sequelae such as scarring and, possibly, MCL)
