# Supplementary material for: Cutaneous leishmaniasis and co-morbid major depressive disorder: A systematic review with burden estimates
Source: PLoS Negl Trop Dis. 2019 Feb 25;13(2):e0007092. doi: 10.1371/journal.pntd.0007092 (PMC6405174; doi:10.1371/journal.pntd.0007092)
Supplement: S6 Appendix — (DOCX) [file pntd.0007092.s006.docx]

**S6 Appendix: Quantifying the burden of co-morbid MDD in cutaneous leishmaniasis**

**Active CL**

Prevalence: 4,320,000^18^

Estimated prevalence of active CL with MDD co-morbidity (20%): 864,000 

|  | **MDD Severity**  **(based on 2010 GBD Study)^22^** | | |
| --- | --- | --- | --- |
|  | **Mild MDD**  **72.7%** | **Moderate MDD**  **16.5%** | **Severe MDD**  **10.8%** |
| **Prevalence (MDD + aCL)** | 628,128 | 142,560 | 93,312 |
| **Disability Weight^75^** | 0.145 | 0.396 | 0.658 |
| **YLDs** | 91,079 | 56,454 | 61,399 |
| **Total YLDs** | 208,932 | | |

**Inactive CL**

Prevalence: 33,883,900

Estimated prevalence of inactive CL with MDD co-morbidity (20%): 6,776,780

|  | **MDD Severity**  **(based on 2010 GBD Study)^22^** | | |
| --- | --- | --- | --- |
|  | **Mild MDD**  **72.7%** | **Moderate MDD**  **16.5%** | **Severe MDD**  **10.8%** |
| **Prevalence (MDD + iCL)** | 4,926,719 | 1,118,169 | 731,892 |
| **Disability Weight^75^** | 0.145 | 0.396 | 0.658 |
| **YLDs** | 714,374 | 442,795 | 481,585 |
| **Total YLDs** | 1,638,754 | | |
